# Serum Prolidase Activity, Oxidant and Antioxidant Status in Nonulcer Dyspepsia and Healthy Volunteers

**DOI:** 10.1155/2013/182601

**Published:** 2013-10-29

**Authors:** Shweta Kumari, Akhilesh Kumar Verma, Sumit Rungta, Rahul Mitra, Ragini Srivastava, Narender Kumar

**Affiliations:** ^1^Department of Biochemistry, Institute of Medical Sciences, Banaras Hindu University, Varanasi 221005, India; ^2^Department of Gastroenterology, Institute of Medical Sciences, Banaras Hindu University, Varanasi 221005, India; ^3^Department of Biochemistry, School of Life Sciences, Dr. B. R. Ambedkar University, Agra 282002, India

## Abstract

*Helicobacter pylori (H. pylori)* infection is associated with increased oxidative stress and serum prolidase activity (SPA) in many diseases. We aimed to observe SPA and oxidative stress in nonulcer dyspepsia (NUD) infected with and without *H. pylori* among eastern Indians. 106 patients with *H. pylori* positive NUD, 82 patients with *H. pylori* negative NUD, and 50 healthy individuals were selected. SPA, total antioxidant capacity (TAOC), and total oxidant status (TOS) were measured with the use of spectrophotometer and an automated measurement method. SPA, TOS, and oxidative stress index (OSI) were significantly higher in patients with *H. pylori* positive than *H. pylori* negative NUD and healthy individuals (all *P* < 0.0001), whereas TAOC was significantly lower (*P* < 0.0001). Nonsignificant, increased SPA (*P* value = 0.6083) and decreased TAOC (*P* value = 0.1186) were observed in patients with *H. pylori* negative NUD than healthy individuals, while increased TOS and OSI were significant (*P* < 0.0001). Weak, nonsignificant correlations were observed between serum prolidase activity and TAOC, TOS, and OSI in *H. pylori* positive cases. Thus, increased SPA along with increased oxidative stress was observed, which seem to be closely associated with *H. pylori* infection. SPA and oxidative stress seem to be used as biomarkers for *H. pylori* infection in NUD.

## 1. Introduction

In 1984, it was first reported that *H. pylori*, a gram negative, spiral shaped, microaerophilic bacterium that colonizes the stomach and is involved in the pathogenesis of duodenal ulceration, gastric cancer, gastric ulceration, and active chronic gastritis [1, 2]. Dyspepsia is a common term used for abdomen pain and complimented with other gastrointestinal symptoms. Nonulcer dyspepsia is characterized with as upper gastrointestinal symptoms of the patients. Nonulcer dyspepsia is also known as functional dyspepsia which found to be associated with *H. pylori* infection [3, 4]. *H. pylori* infection was found to be associated with gastric cancer, peptic ulcer, duodenal ulcer, gastric carcinoma [5–8], and so forth.

Prolidase (EC 3.4.13.9), proline dipeptidase, degraded dipeptides with hydroxyproline or proline as c-terminal amino acid [9, 10]. It participates in collagen metabolism, cell growth, and matrix remodeling [11]. Its activity has been reported in leukocytes, erythrocytes, plasma, and the various organs such as brain, heart, kidney, uterus, thymus, and dermal fibroblasts. Prolidase activity has been reported in various disorders, like osteoarthritis, chronic liver disease, and osteoporosis [12–15].

*H. pylori* infection leads to oxidative stress in gastric mucosa [16]. It has been observed that increased oxidative stress associated with gastroduodenal mucosa inflammation in *H. pylori* infected individuals [17]. It has been reported that *H. pylori* infection induced inflammation and can cause gastric atrophy and it is related to increased oxidative stress [18, 19]. Serum prolidase activity and oxidative status in *H. pylori* infected individuals were studied by Aslan et al. in 2007, among Turkey population [20]. The prevalence of *H. pylori* infection varies from country to country by geographic area, ethnicity, age, race, and socioeconomic status [21, 22] and consists of large diversity of strains [22–27]. Due to its different prevalence and diversity of strains, we aimed to observe oxidative stress and serum prolidase activity in patients with* H. pylori *infected nonulcer dyspepsia among Indian subjects. With serum prolidase activity, we also estimated total antioxidant capacity (TAOC), total oxidant status (TOS), and oxidative stress index (OSI) in the above cases.

## 2. Material and Methods

Analytical grade reagents and chemicals were used in this study.

This study was done in the Department of Biochemistry, Department of Gastroenterology, and Department of Pathology, Institute of Medical Sciences, Banaras Hindu University, Varanasi, India. Institutional ethical committee also approved to carry out this study.

### 2.1. Patients Selection

A total of 238 subjects were selected after informed consent. Out of 238, 188 subjects were patients of nonulcer dyspepsia (NUD) and 50 healthy individuals followed up examination. Among nonulcer dyspeptic patients, 106 *H. pylori* positive and 82 *H. pylori* negative patients were observed. Patients with *H. pylori* positive NUD were considered as cases, while patients with *H. pylori* negative NUD were considered as control-1 group. 50 Healthy individuals were considered as control-2 group. This was done after endoscopy, histopathological examination, and rapid urease test.

### 2.2. Collection and Storage of Samples

Two antral biopsies were obtained during endoscopy. One was fixed with 10% formalin for histopathological examination in the Department of Pathology, and the other was treated with 10% urea solution containing phenol red indicator (0.2%) for the rapid urease test. Venous blood was collected in tubes and stored at 4°C. The serum was separated from the cells by centrifugation at 3000 rpm for 10 min. Serum samples for the measurement of TOS, TAOC, and prolidase activity were stored at −80°C until they were used.

### 2.3. Histopathological Examination

For histopathological and gastritis scoring standard upgraded Sydney criteria were used [28].

### 2.4. Rapid Urease Test

Rapid urease test was used to diagnose *H. pylori* infection. Positive rapid urease test indicated *H. pylori* infection, and negative result indicated negative *H. pylori* infection. A positive reaction was dark pink, and a negative reaction was either a yellow or an orange color. The Media consisted of 10% urea solution—1.5 mL and phenol red indicator (0.2%)—2 drops, pH 6.4–6.8.

### 2.5. Measurement of Total Antioxidant Capacity (TAOC)

For measurement of total antioxidant capacity reagent 1 and 2 were prepared. Reagent 1: 3.17 gm of orthodianisidine dihydrochloride and 0.01764 gm of Fe(NH_4_)_2_(SO_4_)_2_·6H_2_O were dissolved in 1000 mL of Clark and Lubs solution; Reagent 2: 0.641 mL of H_2_O_2_ solution (35%) was diluted to 1000 mL with Clark and Lubs solution. TAOC of serum was measured by the use of an automated measurement method developed by Erel (2004) [29, 30].

### 2.6. Measurement of Total Oxidant Status (TOS)

Reagent 1 and 2 were prepared. Reagent 1: 114 mg of xylenol orange and 8.18 gm of NaCl were dissolved in 900 mL of H_2_SO_4_ solution 25 mM. The final reagent was composed of 150 mM xylenol orange, 140 mM NaCl, and 1.35 M glycerol, pH 1.75; Reagent 2: 1.96 gm of ferrous ammonium sulfate and 3.17 gm of o-dianisidine dihydrochloride were dissolved in 1000 mL of H_2_SO_4_ solution 25 mM. TOS of serum was measured by a method developed by Erel (2004) [29, 30].

### 2.7. Measurement of Oxidative Stress Index (OSI)

Ratio of TOS level to TAOC level was accepted as OSI. OSI values were calculated according to the following formula:

(1) OSI  (arbitrary  unit)  =TOS  (μmol  H2O2  Equiv./L)TAOC  (mmol  Trolox  Equiv./L).

### 2.8. Determination of Prolidase Activity

Chinard's reagent (25 g of ninhydrin was dissolved in 600 mL of glacial acetic acid and 400 mL of 6 mol/L orthophosphoric acid at 70°C), standard proline solution (650 *μ*mol/L solution in 0.45 mol/L trichloroacetic acid), and Gly-l-Pro/Sigma Chemical Co. (94 mmol/L in 0.05 mol/L Tris HCl buffer, pH 7.8, containing 1 mmol of MnCl_2_ per liter) were prepared and stored at 4°C. 

Plasma was diluted six-fold with buffer mixture containing 0.05 mmol/L Tris HCl buffer (pH 7.8) in 2 mmol of MnCl_2_/L and was incubated for 2 hrs at 37°C. After incubation, prolidase reaction was initiated by adding 100 *μ*L of the preincubated mixture to 100 *μ*L of 94 mM Gly-l-Pro solution. After reaction initiation, it was incubated for 30 min at 37°C; reaction was stopped by adding 1 mL of 0.45 mol/L trichloroacetic acid. The released proline was measured by addon of 0.5 mL of supernatant to 2 mL of 1 : 1 mixture of glacial acetic acid : Chinard's reagent and incubated for 10 min at 90°C. A blank and standard were run under the same condition. Instead of the supernatant, blank contains 0.5 mL of 0.45 mol/L trichloroacetic acid. The amount of proline was determined by spectrophotometer at 515 nm. Serum prolidase activity was measured by the method developed by Myara et al., 1984 [13].

### 2.9. Inclusion Criteria

50 healthy individuals and 188**  **consecutive patients with nonulcer dyspepsia who agreed to participate in the study were included in our study. Dyspepsia is characterized by upper abdominal pain or discomfort, nausea, vomiting, bloating, or any other symptom related to the upper gastrointestinal tract. Nonulcer dyspepsia was diagnosed when dyspeptic symptoms were present for at least 4 weeks unrelated to exercise for which no organic lesion, such as peptic ulcer, reflux esophagitis, and gallstones, or any systemic disease, was found to be responsible.

### 2.10. Exclusion Criteria

The following patients were excluded from our study: patients with coexistent medical illness like chronic heart failure, chronic renal failure, diabetes mellitus, and so forth, pregnant females, patients with continued use of nonsteroidal antiinflammatory drugs, patients with history of use of H_2_-receptor antagonist/proton pump inhibitors during the previous 3 months, patients with evidence of any organic gastrointestinal/or hepatobiliary diseases (diagnosed by abdominal ultrasound or upper GI endoscopy), patients that their informed consents are not available, and patients who did not agree on followup at the regular interval. Patients with history of alcohol intake and antioxidant supplement consumption were excluded.

### 2.11. Statistical Analysis

Standard statistical methods were used in this study. Parametric methods were used; a *P* value of less than 0.05 (*P* < 0.05) was considered significant. Mean ± SD was used for data representation. Graph pad and SPSS 16.0 software were used.

## 3. Results

### 3.1. Chronic Inflammation Grading

Most of cases in our study were of moderate inflammation 34 (32.1%) on histopathological examination, while mild inflammation was present in 32 (30.2%) cases. 22 cases (20.8%) had severe inflammation, while 18 (17%) cases had zero grade inflammation of the gastric mucosa. Grading index for control-1 and control-2 of chronic inflammation was zero (Table 1).

### 3.2. Activity Grading (Intensity of Neutrophilic Infiltration)

Activity is denoted by intensity of neutrophilic infiltration. In our study, most of 36 (34%) *H. pylori* positive subjects show moderate neutrophilic infiltration. While 30 (28.2%), 20 (18.9%), and 20 (18.9%) cases showed mild, severe, and no neutrophilic infiltration activity, respectively. Control-1 and control-2 had zero activity grading (Table 1).

### 3.3. Serum Prolidase Activity in Cases and Controls

Serum prolidase activity was found to be higher in *H. pylori *positive subjects (37.91 ± 3.19 mmol min^−1^ L^−1^) as compared to *H. pylori* negative (32.19 ± 3.43 mmol min^−1^ L^−1^) and healthy subjects (31.92 ± 3.10 mmol min^−1^ L^−1^). Therefore, serum prolidase activity was significantly higher in cases as compared to controls (*P* < 0.0001). It was observed that control-1 had increased serum prolidase activity than control-2, but this was nonsignificant (*P* = 0.6083) (Table 2).

### 3.4. Total Antioxidant Capacity in Cases and Controls

Total antioxidant capacity (TAOC) in *H. pylori* positive, *H. pylori* negative, and healthy subjects were 1.39 ± 0.36, 1.79 ± 0.37, and 1.88 ± 0.21 mmol Trolox Eq/L, respectively. Thus, the TAOC was statistically lower in *H. pylori* positive cases than controls (*P* < 0.0001). Control-1 had decreased TAOC than control-2, which was nonsignificant (*P* = 0.1186) (Table 2).

### 3.5. Total Oxidant Status

Total oxidant status (TOS) in cases, control-1, and control-2 were 13.29 ± 1.29, 11.57 ± 1.06, and 10.65 ± 0.21 *μ*mol H_2_O_2_ Equiv./L, respectively. Thus, total oxidant status was significantly higher in cases as compared to controls (*P* < 0.0001). Significantly increased TOS was observed more in control-1 than in control-2 (*P* < 0.0001) (Table 2).

### 3.6. Oxidative Stress Index

OSI was found to be significantly higher in cases (10.25 ± 3.12) as compared to control-1 (6.71 ± 1.47) and control-2 (5.72 ± 0.56) (*P* < 0.0001). Increased OSI was observed more in control-1 than in control-2, which was significant (*P* < 0.0001) (Table 2).

### 3.7. Correlative Observation

Weak, negative, and nonsignificant correlation was observed between serum prolidase activities and TAOC in cases (*r* = −0.131, *P* = 0.181), while weak, positive, and nonsignificant correlation was observed between serum prolidase activity and TOS and OSI (*r* = 0.029, *P* = 0.77, and *r* = 0.107, *P* = 0.277, resp.). This above type of weak, nonsignificant correlation was also observed for controls (Table 3).

## 4. Discussion

For gastritis evaluation, upgraded Sydney system was followed [28]. In general, the inflammation induced by *H. pylori* infection has a chronic active gastritis which means that both lymphocytes and neutrophils infiltrate the mucosa in a characteristic manner. Apart from the inflammatory infiltrate, foci of intestinal metaplasia with atrophy, lymphatic aggregates, and lymphoid follicles were formed, and the foveolar epithelium was replaced by regenerative epithelium with correspondingly reduced mucus secretion [31]. Because it is found throughout the gastric mucosa from pylorus to cardia, *H. pylori* gastritis is usually pangastritis although in severity it may vary considerably between the antrum and corpus. In the present study, majority of the cases (32.0%) had moderate inflammation, while mild and severe inflammation was seen in 30.2% and 20.8% of cases, respectively. On analyzing activity, mild activity observed in 28.2% of cases, while 34.8% and 18.9% of cases had moderate and severe activity, respectively. 18.9% of cases had no activity (Table 1), while in control-1 and control-2, zero grading was observed for both chronic inflammation and neutrophilic infiltration. Thus, *H. pylori* infected nonulcer dyspeptic (NUD) patients seem to have more chronic inflammation and neutrophilic infiltration than *H. pylori* negative NUD and healthy subjects.

TOS, TAOC, and OSI are used as biomarkers of oxidative stress and reflect the redox between oxidation and antioxidation [29]. It is well known that oxidative stress can be defined as an increase in oxidants or a decrease in antioxidant capacity, and various oxidants and antioxidants have additive effects on oxidative stress [32]. Although the concentration of plasma level of oxidants and antioxidants can be measured individually, it may not accurately reflect the oxidative status [33–35]. Here, we observed oxidative stress in the study population by using TOS, TAOC, and OSI. TOS in *H. pylori* infected dyspeptic subjects was found to be higher when compared to *H. pylori* negative subjects with functional dyspepsia and healthy subjects. In Turkey population, TAOC was found to be significantly lower in *H. pylori* positive subjects as compared to control, while oxidative stress index was found to be higher [20]. Among 238 eastern Indian subjects, mean ± SD of TAOC was found to be significantly lower in *H. pylori* positive subjects as compared to *H. pylori*-negative nonulcer dyspeptic and healthy individuals (*P* < 0.001). Oxidative stress index was found to be higher in cases as compared to controls (*P* < 0.001). Here we observed that in *H. pylori* infected NUD as oxidative stress (TOS, OSI) increases, the TAOC value significantly decreases as compared to *H. pylori* negative NUD and healthy subjects. Thus, *H. pylori* infection associated with increased oxidative stress and decreased antioxidant level in nonulcer dyspeptic patients.

Prolidase enzyme activity had been investigated in various disorders. In subjects with liver disease, serum prolidase activity had been showed to increase especially in early stage of fibrosis. It has been suggested that plasma prolidase activity might be useful to evaluate the fibrotic processes in chronic liver disease in human [36]. A study by Horoz et al. from Turkey represented that serum prolidase activity is to be significantly higher in patients with NASH in comparison to controls (*P* = 0.016) [37]. They also found a significant correlation between serum prolidase activity and fibrosis score (*r* = 0.661, *P* < 0.001). Increased serum prolidase activity was well documented in certain cancers like pancreatic cancer, lung carcinoma, breast cancer, Stage 1 endometrial cancer, stomach cancer, ovarian cancer [38–40], and so forth. In our study population, increased serum prolidase activity was found in patients with *H. pylori* positive NUD as compared to patients with *H. pylori *negative NUD and healthy subjects. This is comparable with the results of the study of Aslan et al. (2007) [20], who also observed that prolidase activity is to be higher in patients with *H. pylori* positive subjects with mean serum prolidase activity of 44.11 ± 3.71 U/L against 39.18 ± 5.40 U/L in patients with *H. pylori* negative subjects.

This study concluded that in patients with nonulcer dyspepsia of eastern Indian subjects increased oxidative stress and increased serum prolidase activity may be associated with *H. pylori* infection and their association may help to grant a better understanding regarding the pathogenesis of *H. pylori* infection. Thus, serum prolidase activity may be used as biomarker for *H. pylori* infection in patients with nonulcer dyspepsia. It was also observed that chronic inflammation and neutrophilic infiltration of gastric mucosa are significantly related to *H. pylori* infection in patients with nonulcer dyspepsia.

## Figures and Tables

**Table 1:** Histopathological examination of gastritis in cases, control-1, and control-2 (chronic inflammation and intensity of neutrophilic infiltration); for this scoring standard upgraded Sydney criteria were used [28]. No—numbers, %—percent.

Grading index	Chronic inflammation						Intensity of neutrophilic infiltration					
	For cases		For control-1		For control-2		For cases		For control-1		For control-2	
	No.	%	No.	%	No.	%	No.	%	No.	%	No.	%
Grade 0	18	17.0	82	100	50	100	20	18.9	82	100	50	100
Grade 1 (mild)	32	30.2	Nil	Zero	Nil	Zero	30	28.2	Nil	Zero	Nil	Zero
Grade 2 (moderate)	34	32.0	Nil	Zero	Nil	Zero	36	34.0	Nil	Zero	Nil	Zero
Grade 3 (severe)	22	20.8	Nil	Zero	Nil	Zero	20	18.9	Nil	Zero	Nil	Zero

Total	106	100	82	100	50	100	106	100	82	100	50	100

**Table 2:** Representation of serum prolidase activity, total antioxidant capacity, total oxidant status, and oxidative stress index in the cases, control-1, and control-2. Data is represented as mean ± standard deviation with their respective units. *P*-value of less than 0.05 showed significant changes. Cases have significantly more increased serum prolidase activity, total oxidant status, and oxidative stress index than control-1 and control-2, while total anti-oxidant capacity significantly decreased.

Subjects/statistical parameters	Number of subjects/condition	Serum prolidase activity (mmol min^−1^ L^−1^, mean ± SD)	Total antioxidant capacity (mmol Trolox Eq/L, mean ± SD)	Total oxidant status (*μ*mol H_2_O_2_ Eq/L, mean ± SD)	Oxidative stress index (arbitrary unit, mean ± SD)
Cases	106	37.91 ± 3.19	1.39 ± 0.36	13.29 ± 1.29	10.25 ± 3.12
Control-1	82	32.19 ± 3.43	1.79 ± 0.37	11.57 ± 1.06	6.71 ± 1.47
Control-2	50	31.92 ± 3.10	1.88 ± 0.21	10.65 ± 0.21	5.72 ± 0.56

Total	238	—	—	—	—

*P*-value (two-tailed)	Cases versus control-1	≤0.0001	≤0.0001	≤0.0001	≤0.0001
	Cases versus control-2	≤0.0001	≤0.0001	≤0.0001	≤0.0001
	Control-1 versus control-2	Equal to 0.6083	Equal to 0.1186	≤0.0001	≤0.0001

**Table 3:** Representation of correlation of serum prolidase activity and TAOC, TOS, and OSI for the cases, control-1, and control-2. Correlation coefficients of ≤0.01 are considered as significant correlation. Negative sign for correlation coefficients indicated that serum prolidase activity increased with decrease in TAOC in cases, control-1, and control-2, while positive values indicated that serum prolidase activity increased with increase in TOS and OSI in respective cases, control-1, and control-2. In our above correlation study, all correlations have non-significant values.

Statistics/correlative parameters	Serum prolidase activity versus TAOC			Serum prolidase activity versus TOS			Serum prolidase activity versus OSI		
	Cases	Control-1	Control-2	Cases	Control-1	Control-2	Cases	Control-1	Control-2
Pearson correlation coefficients									
Coefficients, *r*	−0.131	−0.013	−0.170	0.029	0.093	0.029	0.107	0.087	0.152
Sig. (two-tailed), *P*	0.181	0.907	0.238	0.770	0.407	0.840	0.277	0.437	0.292
